# Leg joint power output during progressive resistance FES-LCE cycling in SCI subjects: developing an index of fatigue

**DOI:** 10.1186/1743-0003-5-14

**Published:** 2008-04-26

**Authors:** Stephenie A Haapala, Pouran D Faghri, Douglas J Adams

**Affiliations:** 1 Functional Performance Laboratory, Department of Allied Health Sciences, University of Connecticut, Storrs, CT, USA; 2 Biomedical Engineering Program School of Engineering, University of Connecticut, Storrs, CT, USA; 3 Department of Orthopaedic Surgery, University of Connecticut Health Center, Farmington, CT, USA

## Abstract

**Background:**

The purpose of this study was to investigate the biomechanics of the hip, knee and ankle during a progressive resistance cycling protocol in an effort to detect and measure the presence of muscle fatigue. It was hypothesized that knee power output can be used as an indicator of fatigue in order to assess the cycling performance of SCI subjects.

**Methods:**

Six spinal cord injured subjects (2 incomplete, 4 complete) between the ages of twenty and fifty years old and possessing either a complete or incomplete spinal cord injury at or below the fourth cervical vertebra participated in this study. Kinematic data and pedal forces were recorded during cycling at increasing levels of resistance. Ankle, knee and hip power outputs and resultant pedal force were calculated. Ergometer cadence and muscle stimulation intensity were also recorded.

**Results:**

The main findings of this study were: (a) ankle and knee power outputs decreased, whereas hip power output increased with increasing resistance, (b) cadence, stimulation intensity and resultant pedal force in that combined order were significant predictors of knee power output and (c) knowing the value of these combined predictors at 10 rpm, an index of fatigue can be developed, quantitatively expressing the power capacity of the knee joint with respect to a baseline power level defined as fatigue.

**Conclusion:**

An index of fatigue was successfully developed, proportionalizing knee power capacity during cycling to a predetermined value of fatigue. The fatigue index value at 0/8^th ^kp, measured 90 seconds into active, unassisted pedaling was 1.6. This indicates initial power capacity at the knee to be 1.6 times greater than fatigue. The fatigue index decreased to 1.1 at 2/8^th ^kp, representing approximately a 30% decrease in the knee's power capacity within a 4 minute timespan. These findings suggest that the present cycling protocol is not sufficient for a rider to gain the benefits of FES and thus raises speculation as to whether or not progressive resistance cycling is an appropriate protocol for SCI subjects.

## Background

Functional electrical stimulation-leg cycle ergometry (FES-LCE) has been considered an effective muscle exercise therapy for spinal cord injured (SCI) individuals. Disuse associated with paralysis causes morphological and metabolic changes, inducing the conversion of type I to type II, or slow-twitch to fast-twitch muscle fibers [1,2]. Regular implementation of FES-LCE has helped paralyzed muscle revert back to the behavior and properties closer to healthy muscle as well as to increase muscle strength, increase resistance to fatigue, decrease contraction time, maintain bone and muscle integrity, improve lower extremity circulation and relieve and prolong the onset of secondary conditions associated with spinal cord injury [1,3-9]. A primary objective for improving FES-LCE is to maximize riding time, which increases the cardiovascular benefits of the workout and improves stamina. To do so, it is important to develop protocols that delay the onset of fatigue during cycling as well as to assess present levels of fatigue so that appropriate adjustments in FES can be made to maintain pedaling efficiency. Many of the stimulation protocols presently implemented in FES-LCE may accelerate the onset of fatigue due to their "one size fits all" paradigm of stimulation. Fatigue assessment may help in customizing the FES-LCE stimulation protocol to each rider; allowing for a more effective match between a subject's needs and subsequent muscle stimulation.

Several approaches have been taken to monitor force generation in able-bodied and SCI subjects, and improve fatigue in paralyzed muscle [10-13]. Electromyography (EMG) has been used to assess fatigue by evaluating the decreased muscle force and related changes in the root mean square (RMS) of the EMG amplitude and shifts in the median frequency of the EMG power spectrum during electrical stimulation [12,13]. A common obstacle in these studies was the production of a reliable EMG signal without the presence of a stimulation artifact. Other studies have investigated the general effect of different stimulation protocols on fatigue generation in paralyzed muscle as well as effects on cycling performance [14,15]. During an investigation of the effects of stimulation protocol on thenar muscle force generation, Thomas revealed that variable rate stimulation produced slightly higher muscle forces than a constant rate protocol. However, SCI subjects fatigued quicker than able-bodied subjects, regardless of the stimulation protocol used [14]. Eser, et al found that modulating the frequency of applied stimulation from 30 Hz to 60 Hz increased power output at the ergometer pedal during submaximal cycling [15]. Unfortunately, prolonged exposure to higher stimulation frequencies has been linked to rapid muscle fatigue and therefore is not commonly implemented in FES-LCE of SCI subjects [16].

Computational and mathematical models have also been implemented to predict and estimate joint mechanics and stimulated muscle's force generating capacity [17-20]. Giat et al, developed a musculotendon model of paralyzed quadriceps muscle that incorporated fatigue to predict muscle force generated during continuous stimulation. The resulting force profiles closely matched muscle force decay observed experimentally, but could not be generalized due to subject sample size [17]. Trumbower et al developed a Probably Approximately Correct (PAC) model which successfully predicted the strength capacity of paralyzed thigh muscles of potential ergometer riders, producing results comparable to dynamic strength values [18]. This study demonstrated a possible approach to the individualization of FES cycling protocols. However, the muscles were evaluated on the basis of "Fatigue" or "No-Fatigue". The actual level of fatigue present in the muscles was not quantified.

Other studies have applied existing mechanical principles in order to quantify muscular changes within the leg system [21-26]. Using recorded pedal forces, joint kinematics and anthropometric data, Ericson calculated the instantaneous power output of the hip, knee and ankle in able-bodied subjects during ergometer riding for different levels of resistance and cadence. The expression moment = force*distance uses calculated joint moment to quantify force generation in the muscle belly [23]. This mechanical approach of determining joint moment from actual subject data quantifies changes in muscle force that EMG and modelling do not. However, few studies involving SCI subjects have focused on mechanical changes of the entire leg system during ergometer cycling. This information may be potentially insightful to the understanding of fatigue onset.

The purpose of this study was to investigate the biomechanics of the hip, knee and ankle during a progressive resistance cycling protocol in an effort to detect and measure the presence of muscle fatigue. Joint power output, a primary parameter of interest, can be influenced by muscle force generation as well as joint flexion/extension. Studies have suggested that the quadriceps muscles are the primary source of force generation during forward cycling [18,20,23,27]. Additionally the knee is the only freely moving joint examined in this study. Due to its proximity to both the hamstring and quadriceps muscle groups and ability to move without constraint, the knee was the only joint to accurately reflect changes in power output as a result of changes in both parameters. Therefore, we hypothesized that knee power output is an effective predictor of lower limb fatigue and can be used to develop a fatigue index. Since the complex nature of FES-LCE cycling exceeds the scope of this paper, it is hoped that this index may act as a diagnostic tool in order to modify those factors which influence FES cycling and ultimately lengthen cycling time in SCI subjects.

## Methods

### Subjects

Six spinal cord injured subjects participated in the study. All subjects had previous experience with FES cycle ergometry, were between the ages of twenty and fifty years old, and possessed either a complete or incomplete spinal cord injury at or below the fourth cervical vertebra. Two subjects had incomplete injuries, four had complete injuries. All subjects signed a consent form, explaining the terms and conditions of the study in agreement with the Institutional Review Board of the University.

### FES-LCE system – ERGYS I™

**Figure 1:**
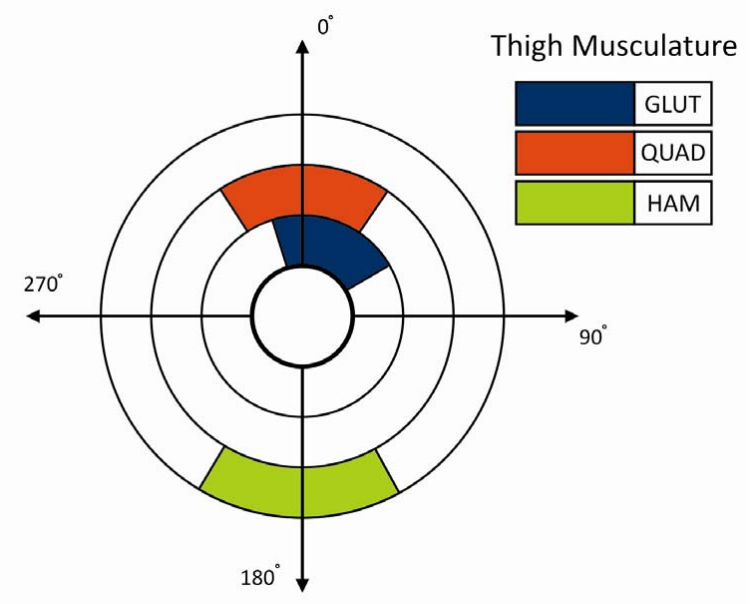
**Crank Angle Diagram**. Pre-programmed sensors were used to stimulate the appropriate muscles during specific phases of cycle rotation, using a sinusoidal, biphasic waveform with a pulse duration of 500 μsec, a phase duration of 1000 μsec and frequency of 50 Hz. Quadriceps and gluteus stimulation was initiated prior to reaching top dead center (TDC) during leg extension. Hamstring stimulation was applied through bottom dead center (BDC), during leg flexion. Stimulation was provided through individual stimulation channels.

**Figure 2:**
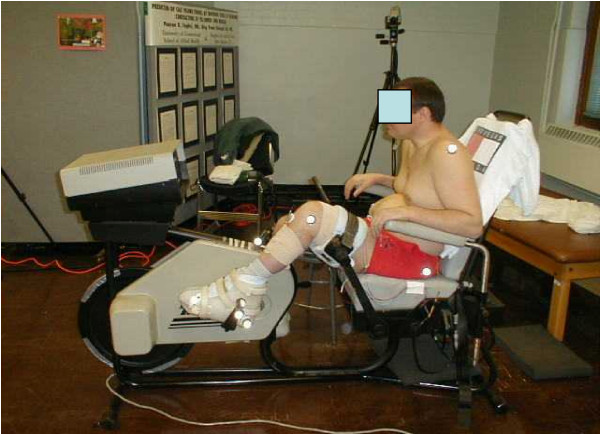
**Complete Setup of the FES-LCE System**. The subject is seated and secured within the FES-LCE system. The feet are placed in boots that are fixed to the ergometer pedal. The thighs are secured with Velcro straps, which help to maintain movement in the sagittal plane only. Seat depth is adjusted so that the subject's leg does not fully extend when the ergometer pedal is 110° with respect to TDC. Reflective markers were placed at the shoulder, hip, knee, ankle, toe, heel of boot, pedal spindle, pedal force sensor, ergometer crank center, and ergometer frame.

The ERGYS I™ (Therapeutic Alliances^®^, Inc., Fairborn, OH) semi-reclined cycle ergometer was used in this study. Resistance was produced by increasing the tension of a friction-induced band applied to the perimeter of the flywheel and secured to the ergometer frame. Tensions required to produce ergometer power outputs of 0, 6.25 and 12.5 Watts were determined assuming a constant cadence of 50 rpm. 6.25W of ergometer power is equivalent to a resistance of 1/8^th ^kp. A digital speedometer was attached to the front of the ergometer, allowing the subject to monitor their current cadence levels. If cadence levels fell below 10 rpm, stimulation was terminated and subjects were assisted in a passive cycling cool-down. Stimulation was supplied by the ergometer in the form of a sinusoidal, biphasic waveform with a pulse duration of 500 μsec, a phase duration of 1000 μsec and frequency of 50 Hz. Maximum stimulation intensity was 140 mA. Since each muscle contracted during a different phase of cycle rotation, pre-programmed sensors were used to stimulate appropriate muscle groups at specific crank angles (Figure 1). Seat depth of the ergometer was adjusted horizontally for each rider so that the rider's knee was not fully extended when the pedal reached an angle of 110° with respect to top dead center (TDC). The subject's feet were secured within boots attached to the ergometer pedal and the thighs were secured with Velcro straps to restrict movement perpendicular to the sagittal plane. A complete setup of the subject in the FES-LCE system can be viewed in Figure 2.

**Figure 3:**
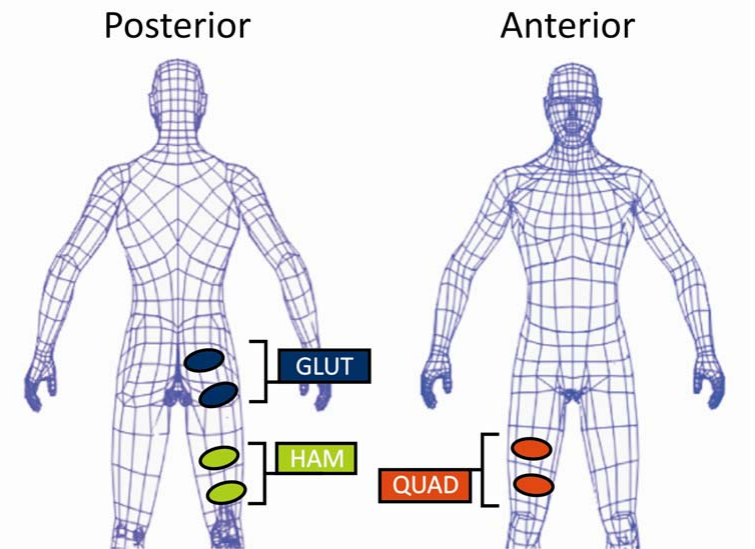
**Electrode Placement**. Approximate locations for electrode placement superficial to QUAD, HAM, and GLUT, muscles. Active and ground electrodes were spaced approximately 4 cm apart. The QUAD ground electrode was placed approximately 5 cm from the patellar apex, HAM ground electrode was placed approximately 5 cm from the knee crease, and GLUT ground electrode placed along the gluteal fold. To maintain consistency, electrode placement was performed by the same person for all subjects.

Stimulation was delivered to the quadriceps, hamstrings and gluteus maximus muscles of each leg using two oval, 2.5" × 3.25"self adhesive surface electrodes for each muscle group, figure 3. Each active/ground electrode set was independently stimulated and grounded, eliminating the potential for co-contraction. Surface electrodes were arranged so that the quadriceps ground electrode was placed a distance of approximately 6 cm superior to the patella. The active electrode was placed approximately 10 cm superior and slightly lateral so that it rested over the muscle belly of the rectus femoris and vasti. The hamstring ground electrode was placed a distance of approximately 6 cm superior to the posterior crease of the knee joint; situated approximately over the semitendinosus and short head of the femoral bicep. The active electrode was placed a distance of 10 cm superior so that the muscle bellies of the semitendinosus, semimembranosus and long head of the femoral biceps were covered. The gluteus maximus ground electrode was placed at the gluteal fold. The active electrode was placed approximately 4 cm superior and anterior so as to rest on the muscle belly of the gluteus maximus. To maintain consistency, electrode placement was performed by the same person for all subjects.

### Kinematic data

**Figure 4:**
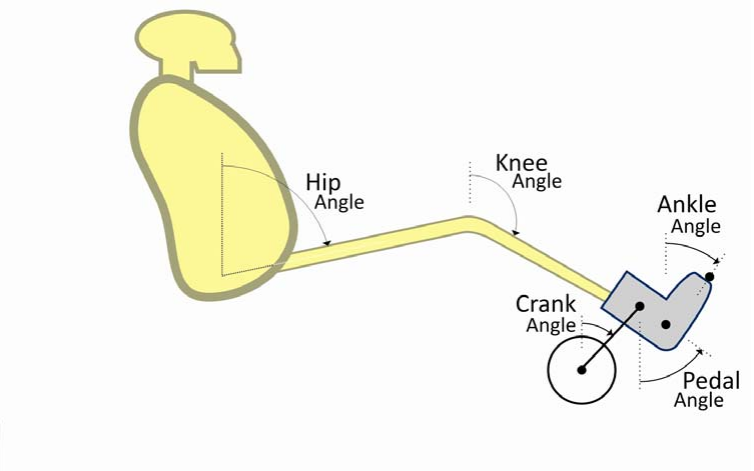
**Joint Angle Measurements**. Angular displacement of the hip, knee and ankle were calculated with respect to vertical. Crank angle was measured with TDC corresponding to 0°. Pedal angle considered angular changes in the pedal spindle-boot heal plane with respect to vertical. Values for hip, knee and ankle power outputs were averaged over the last 10 completed rotations at each resistance level.

**Figure 5:**
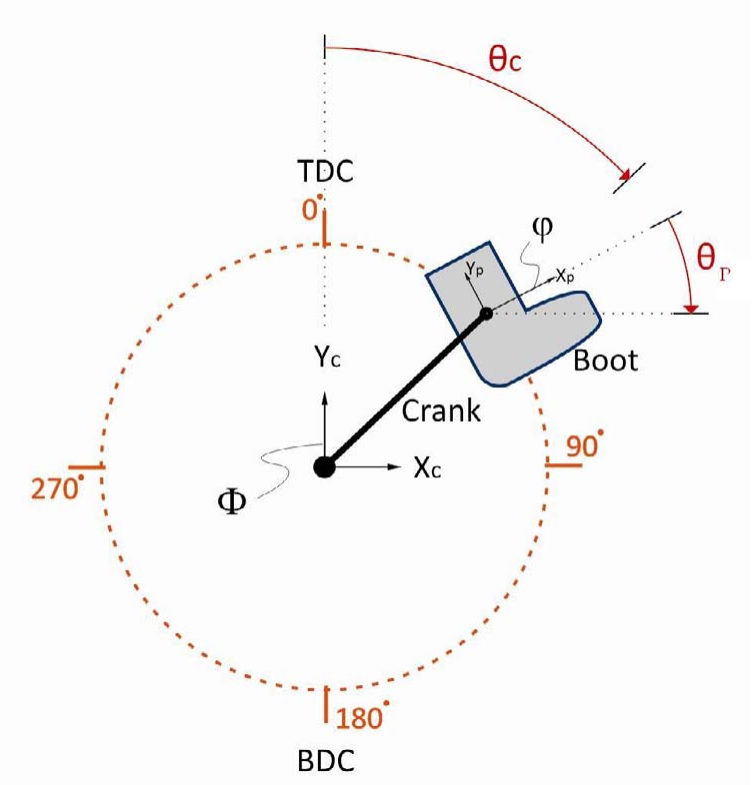
**Geometric Trajectory of the Boot-Pedal System**. Definitions of top dead center (TDC), bottom dead center (BDC), and reference frames for the boot-pedal (ϕ; X_p_, Y_p_) and ground (Φ; X_c_, Y_c_). θ_p _corresponds to boot-pedal angle relative to the horizontal and θ_c _corresponds to crank angle relative to the vertical axis. Leg flexion occurred from 110°–290°, leg extension occurred from 290°-110° of the crank angle.

Adhesive reflective markers were placed over the humeral head of the shoulder, approximately 1 cm anterior and superior to the tip of the greater trochanter, at the center of the lateral femoral epicondyle, the lateral tip of the malleolus, and on the lateral side of the ergometer boot at the approximate location of the fifth metatarsal. Additional markers were placed at the center of the pedal spindle, on the lateral side of the pedal force sensor, at the heal of the ergometer boot, and the ergometer crank center, figure 2. A reference was placed on the frame of the ergometer, vertically aligned with the crank center. The reflective markers were illuminated using a flood light and continuously video recorded, using the image from a video camera oriented perpendicular to the rider's sagittal plane. A video-based motion capture software system (Peak Motus^® ^System, Peak Performance Technologies Inc., Denver, CO) was used to measure the displacement of the crank tip, hip, knee, and ankle joints during cycling (Figure 4). Displacement data were measured at a frequency of 60 Hz. The kinematic data were filtered using a 5^th^-order, zero-lag, low pass Butterworth filter with a cutoff frequency of 4 Hz. All data were expressed as a function of crank angle. One complete rotation was the trajectory of the crank tip as it moved from TDC to bottom dead center (BDC) and back to TDC (Figure 5). All calculations were averaged over the last 10 rotations completed at each resistance level to represent the steady-state values of each parameter of interest.

### Kinetic data

A 4-pin, triax, ICP^® ^piezo-electric force transducer (PCB Piezotronics, Inc., NY) with a full-scale measurement range (45 to 22 k N compression, 2200 N tension) was mounted underneath the boot of the right ergometer pedal to record pedal forces in normal and tangential directions with respect to the pedal plane. The force data were collected at a frequency of 180 Hz using a LABView program (National Instruments Corp^®^, Austin, TX), developed for the study. A synchronizing signal was transmitted to the kinematic video recording at the onset of pedal force acquisition, allowing visual synchronization of the pedal force and video-recorded kinematic data.

### Experimental protocol

A progressive intensity test was conducted in order to investigate stimulation intensity, joint power outputs, cadence, resultant pedal force (RPF) and resistance as possible indicators of fatigue. In the context of this test, fatigue was defined as the point when the subject could no longer cycle at or above 10 rpm. The subjects were passively pedalled through a 2 minute warm-up without stimulation. Following the warm-up, stimulation intensity was applied so that subjects maintained a constant cadence of 50 rpm throughout the exercise. 50 rpm was chosen at the target cadence since this was the cadence at which the tension of the friction belt was determined in order to equal 6.25W or 1/8^th ^kp. Resistance was then increased by 1/8^th ^kilo-pound (kp) (6.25 W) every two minutes. A gradient increase of 2 minutes was chosen to minimize muscular fatigue, but still be considered long enough to achieve steady-state conditions. Ninety seconds after each resistance adjustment, force data were recorded for 30 seconds. Stimulation intensity (mA) and cadence were also recorded at this time. 140 mA was considered 100% stimulation. The subject's heart rate, blood pressure and pulse oxygen concentration (SPO2) were also recorded to ensure that the subject maintained normal metabolic behaviours. Resistance was increased only to a level that each subject felt comfortable. If a rider's cadence dropped below 10 rpm, stimulation was automatically terminated and the subject was passively cycled through a 2-minute cool-down.

### Data analysis

#### Kinematic data

Reflective marker displacement was calculated using Peak Motus™ software. The data points were scaled and filtered as described previously. Corresponding angular velocity and acceleration were then calculated using Matlab^® ^software by taking the first and second derivatives of segment rotations, respectively.

#### Kinetic data

Free body diagrams of the foot, shank and thigh were constructed in order to calculate the forces and torques produced at the ankle, knee, and hip joints, respectively. Pedal force, crank angle, segment mass, joint kinematics and anthropometric data were used to calculate resultant joint forces and joint moments of force. All equations were developed with reference to Hull and Jorge's model for biomechanical analysis of bicycle pedaling [10]. Moments of inertia and centers of gravity were calculated using Winter's anthropometrical table [28]. Instantaneous joint power output was calculated using the following equation:

*P *= *M *× *ω*

Where: P = power (W)

M = joint moment of force (N-m)

*ω *= joint angular velocity (rad/sec)

Resultant pedal force (RPF), as identified by Brown and Jensen [11], can be expressed as the vector sum of muscle forces, gravitational and inertial forces that contribute to the contact force measured at the pedal. This value was calculated using the following equation:

$$RPF=\sqrt{F_{Px}^{2}+F_{Py}^{2}}$$

Where: RPF = Resultant pedal force (N)

*F*_*Px*,*y *_= Pedal forces in the x- and y-directions, with respect to the global sagittal plane, respectively (N)

A one-way analysis of variance (ANOVA) was performed in order to evaluate the difference in mean cadence, mean stimulation intensity, mean joint power outputs, total power output and mean resultant pedal force (RPF) with increasing resistance for the subject group as a whole. A Tukey test was used as the post-hoc comparison. Statistical significance was set at p = 0.05. A Pearson product moment correlation was also performed in order to determine the strength of the linear relationship between each of these seven variables. Those variables possessing a strong or moderate correlation with a joint power output were entered into a multivariable regression to investigate the prediction power of these variables on joint power output. A strong correlation was considered a R coefficient of at least 0.8. A moderate correlation was considered a coefficient of at least 0.5. All statistical analyses were carried out using SPSS™ 13.0 software.

## Results

The highest level of resistance completed varied between subjects. Highest completed levels of resistance were: 1/8^th ^kp, 2/8^th ^kp (2 subjects), 3/8^th ^kp, 4/8^th ^kp, and 6/8^th ^kp. Data from the five subjects that completed progressive cycling through 2/8^th ^kp were included in the statistical analyses. Mean and standard deviations were calculated for cadence, stimulation intensity, ankle power output (APO), knee power output (KPO), hip power output (HPO) and resultant pedal force (RPF). (Table 1). Stimulation intensity was expressed as a percentage of maximum intensity, 140 mA. Mean APO and KPO decreased with increasing resistance. Mean stimulation intensity, HPO and RPF were found to increase with increasing resistance. (Table 1)

**Table 1:** Group statistics. Mean and standard deviation for cadence, stimulation intensity, ankle power output, knee power output, hip power output and resultant pedal force (RPF) at each level of resistance.

Resistance (W)	Cadence (rpm)	Stim Intensity (mA)	Ankle PO (mW)	Knee PO (mW)	Hip PO (mW)	RPF (N)
0.0 (n = 5)	48.2 ± 1.90	66.6 ± 20.3	38.8 ± 10.0	120.0 ± 60.0	-88.5 ± 30.0	22.6 ± 5.0
6.25 (n = 5)	48.0 ± 1.70	98.6 ± 26.9	38.1 ± 10.0	98.4 ± 40.0	-20.4 ± 60.0	23.5 ± 4.10
12.5 (n = 5)	41.0 ± 9.70	116.2 ± 28.1	24.5 ± 20.0	26.2 ± 100.0	27.1 ± 30.0	25.03 ± 5.13

The strongest linear correlations existed between: 1) APO and KPO (r = .89), 2) APO and cadence (r = .86), 3) stimulation intensity and KPO (-.84), 4) cadence and KPO (r = .82) and 5) resistance and HPO (r = .80). Moderate correlations were observed between 1) stimulation intensity and resistance (r = .66), 2) stimulation intensity and APO (r = -.66), 3) RPF and KPO (r = .56), 4) cadence and stimulation intensity (r = -.55) and 5) resistance and KPO (r = -.54) (Table 2).

**Table 2:** Pearson Correlation Table for resistance, cadence, stimulation intensity, APO, KPO, HPO and RPF.

		Resistance	Cadence	Stimulation Intensity	Ankle Power Output	Knee Power Output	Hip Power Output	Resultant Pedal Force
Resistance	Pearson Correlation	1						.227
	Sig. (2-tailed)							.417
	N	15						15
Cadence	Pearson Correlation	-.476	1					.285
	Sig. (2-tailed)	.073						.304
	N	15	15					15
Stim	Pearson Correlation	.662(**)	-.553(*)	1				-.356
	Sig. (2-tailed)	.007	.033					.193
	N	15	15	15				15
APowerOut	Pearson Correlation	-.346	.864(**)	-.658(**)	1			.484
	Sig. (2-tailed)	.207	.000	.008				.067
	N	15	15	15	15			15
KPowerOut	Pearson Correlation	-.537(*)	.818(**)	-.837(**)	.889(**)	1		.559(*)
	Sig. (2-tailed)	.039	.000	.000	.000			.030
	N	15	15	15	15	15		15
HPowerOut	Pearson Correlation	.804(**)	-.184	.438	-.108	-.320	1	.163
	Sig. (2-tailed)	.000	.512	.102	.701	.245		.562
	N	15	15	15	15	15	15	15
ResPedalForce	Pearson Correlation	.227	.285	-.356	.484	.559(*)	.163	1
	Sig. (2-tailed)	.417	.304	.193	.067	.030	.562	
	N	15	15	15	15	15	15	15

** Correlation is significant at the 0.01 level (2-tailed).

* Correlation is significant at the 0.05 level (2-tailed).

### Stimulation intensity & hip power output

**Figure 6:**
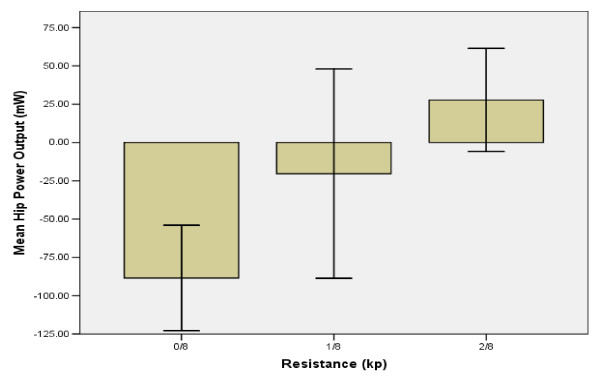
**Resistance vs. Hip Power Output**. The height of the bars represent the mean hip power output for the subject group at each resistance level. Negative values represent power absorption, where the direction of force generation opposes the direction of cycling motion. Positive values indicate power exertion, where the direction of force generation and cycling motion coincide. The range between the two tails indicates the standard error of the mean. Standard error was set at 95%.

Final values of stimulation intensity and HPO were significantly higher than initial values at 0/8^th ^kp (116.2 ± 28.1 mA vs. 66.6 ± 20.3 mA, p = 0.03, and 27.1 ± 30 mW vs. -88.5 ± 30 W, p < 0.01, respectively). Stimulation intensity increased 23% between the first two levels of resistance and an additional 13% by the final resistance level of 2/8^th ^kp. HPO increased 59% from 0/8^th ^to 1/8^th ^kp, and increased by 41% at 2/8^th ^kp. 100% HPO corresponded to the maximum power output observed at 2/8^th ^kp. (Figure 6)

### Cadence

Mean cadence remained close to the target cadence of 50 rpm for the first two resistance levels, experiencing only a slight decrease of 0.2 rpm (Table 1). Mean cadence decreased by 7 rpm between 1/8^th ^and 2/8^th ^kp. Changes observed in mean cadence were not statistically significant between any of the resistance levels. (p = 0.12)

### Ankle & knee power output

**Figure 7:**
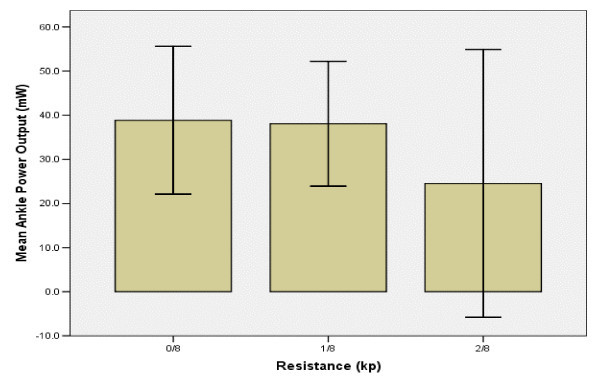
**Resistance vs. Ankle Power Output**. The height of the bars indicate mean ankle power output for the subject group with increasing resistance. The error bars explain 95% of the standard deviation from the mean value. Mean APO remained nearly constant from 0/8^th ^to 1/8^th ^kp, suggesting cadence to be the source of APO production. The observed decrease in ankle power output is likely attributed to the absence of lower leg stimulation.

**Figure 8:**
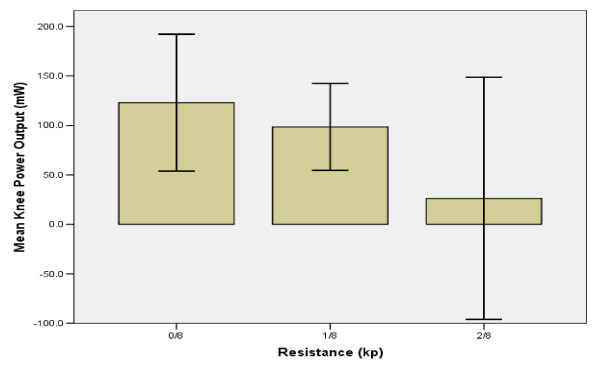
**Resistance vs. Knee Power Output**. Mean KPO decreased with increasing resistance. Since cadence remained nearly constant between 0/8^th ^and 1/8^th ^kp, the decrease in KPO is attributed to a decrease in force generation in the quadriceps and/or hamstring muscles.

Mean APO and KPO did not change significantly with increased resistance. Mean APO decreased slightly from 0/8^th ^to 1/8^th ^kp, and decreased by 35% by 2/8^th^kp (Figure 7). KPO decreased by 20% between the first two levels of resistance and decreased another 59% between 1/8^th ^and 2/8^th ^kp (Figure 8).

### Resultant pedal force

**Figure 9:**
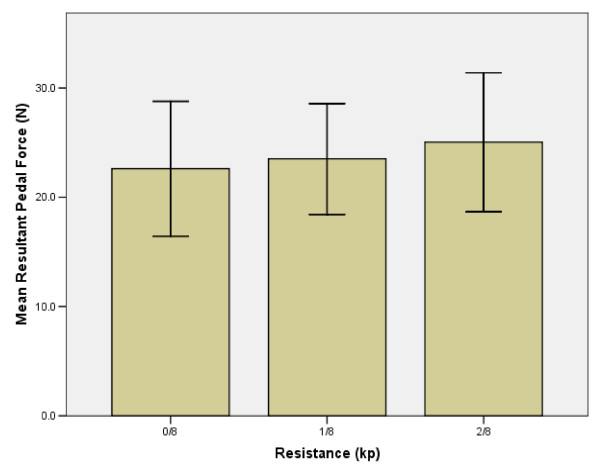
**Resistance vs. Resultant Pedal Force**. RPF quantitatively represents the contribution of muscle, inertial and gravitational forces that contribute to contact force measured at the ergometer pedal. [11] The near constant cadence over the first two resistance levels suggests that the inertial forces of the thigh, shank and foot were responsible for maintaining cadence. Additionally, the decrease in hip power absorption at 1/8^th ^kp possibly translated to an increase in RPF. At 2/8^th ^kp, the only increase observed was in hip power output. Inertial forces would have decreased due to the decrease in ergometer cadence. The subsequent increase in RPF at this time suggests that the increased muscle forces about the hip are primarily responsible for this change.

**Figure 10:**
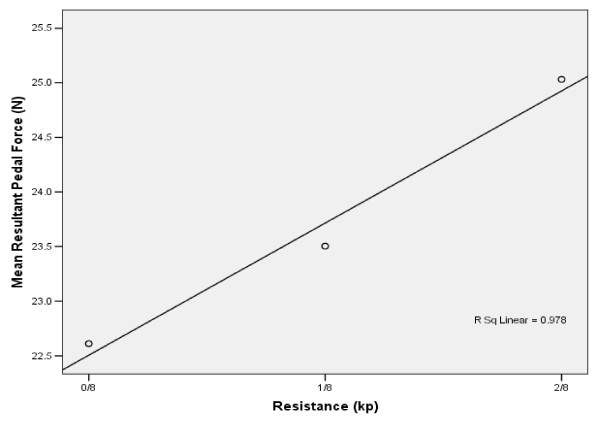
**Resistance vs. Mean Resultant Pedal Force**. The mean RPF values calculated for the subject group as a whole were found to have a linear relationship with resistance. Mean RPF increased with resistance, r^2 ^= 0.98.

RPF did not change significantly with increasing resistance. From 0/8^th ^to 1/8^th ^kp and 1/8^th ^to 2/8^th ^kp, RPF increased by 3.5% and 6.1%, respectively (Figures 9, 10).

### Fatigue indices

Regression lines for APO and KPO were developed to calculate indices of fatigue. APO was predicted by cycling cadence (R^2 ^= 0.75, p < 0.001). APO = 0.002C - 0.074. Considering a cadence of 10 rpm as fatigue, ankle fatigue occurs at -0.054 W. As a result, the index of fatigue represents the proportion of power present at the ankle joint with respect to fatigue:

$$\frac{APO_{M}-APO_{F}}{\left|APO_{F}\right|}=\text{Index of fatigue for Ankle}$$

Where *APO*_*M *_is ankle power output measured during cycling. As APO approaches fatigue, the index will approach 0.

A multiple linear regression of cycling cadence (C), stimulation intensity (S) and RPF (R) provided a significant predictor of KPO = 0.006C - 0.002S + 0.004R - 0.17 (R^2 ^= 0.94, p < 0.05). At fatigue, cadence and stimulation intensity were defined as 10 rpm and 100%, respectively. Since RPF does not have a definitive point of fatigue that can be measured, the mean value of RPF at 2/8^th ^kp was used.

Incorporating this value into the regression equation for KPO indicates that if fatigue occurs at 2/8^th ^kp, KPO will be -209.88 mW, absorbing power. The index of fatigue developed for KPO presents the proportion of existing KPO with respect to fatigue:

$$\frac{KPO_{M}-KPO_{F}}{\left|KPO_{F}\right|}=\text{ Index of fatigue for Knee}$$

where *KPO*_*M *_is measured during cycling and *KPO*_*F *_is the value at fatigue.

Therefore, as KPO approaches fatigue, the index will approach a value of 0. The fatigue indices were applied to the group mean APO and KPO values (Table 3). Application of the ankle fatigue index indicates that at 0/8^th ^kp, the ankle joint has 1.7 times as much power than levels calculated for fatigue. Additionally, the ankle power index decreases to 1.45 by 2/8^th ^kp. The knee fatigue index at 0/8^th ^kp was 1.6 times greater than fatigue. The decrease in knee fatigue index to 1.1 at 2/8^th ^kp indicates a greater reduction in knee power capacity than that experienced at the ankle. The regression equation for HPO was HPO = 0.009R - 0.085 (p < 0.001), whereby resistance was the only predictor (R^2 ^= 0.65). Therefore, no factors define HPO at fatigue and an index of hip fatigue cannot be developed.

**Table 3:** Calculated Fatigue Index Values. Calculated fatigue index values for the ankle and knee at each level of resistance.

Resistance	Ankle Fatigue Index Values	Knee Fatigue Index Values
0/8^th ^kp	1.72	1.59
1/8^th ^kp	1.71	1.47
2/8^th ^kp	1.45	1.12

## Discussion

### Overview

The main findings of this study were: (1) APO and KPO decreased with increasing resistance whereas HPO increased with resistance; (2) cadence, stimulation intensity, and RPF were significant predictors of KPO; and (3) knowing the value of these predictors at 10 rpm, an index of fatigue can be developed. This index quantitatively expresses the power generated at the knee joint with respect to a baseline power level defined as fatigue.

### Ankle

The absence of lower leg stimulation in this exercise suggests that changes observed in APO must be attributed to changes in ankle angular velocity. Studies have suggested that plantar-flexion of the ankle joint during late recovery phase in both able-bodied and SCI subjects was a direct result of boot-pedal inertial forces [29]. Such inertial forces would be augmented by changes in cadence [30]. The subsequent changes in mean APO observed in this exercise demonstrated a strong link to changes in cadence, supporting the identification of cadence as the only significant predictor of APO. The inclusion of lower leg stimulation to such muscles as the tibialis anterior, soleus and gastrocmenius would likely improve cycling performance and potentially increase the circulatory and cardiovascular benefits to the user [27,31-34].

### Knee

The increase in stimulation intensity and subsequent decrease in knee power by 1/8th kp, 3.5 minutes into cycling, are most likely due to premature fatiguing of the quadriceps and/or hamstrings [12-14]. However, the exact muscle group that fatigued cannot be identified since all muscle groups received identical increases in stimulation when cadence dropped below target. Despite potential fatigue, 86% of the power generated within the leg was produced at the knee at 1/8^th ^kp. Research involving able-bodied subjects has reported the quadriceps to remain active over the largest range of the cycling period [23]. The quadriceps muscles also exhibit the greatest strength in both paralyzed and able-bodied riders and make a larger contribution to forward cycling than the hamstrings and gluteus muscles [18,20,23,27]. Therefore, the quadriceps may be primarily responsible for knee power output in this exercise, but additional studies would be required to confirm this conclusion. Cadence-dependent inertial forces may have contributed to knee power output as well. The strong correlation between ankle and knee power outputs (r = 0.889, Table 2) may reflect the boot-pedal's contribution to knee joint moment. Also, moments created by thigh and shank inertial forces may have further increased knee power output. The decrease in KPO at 2/8^th ^kp was likely due not only to a decrease in knee angular velocity, but also from a decline in inertial force associated with decreased cadence, subsequently compromising knee joint moment.

### Hip

The hip absorbed power for the first two resistance levels (Tables 1 and 3). Power absorption is specified by a negative power value and indicates that the direction of muscle force production opposes the direction of crank motion. Power absorption may result from fatigue, improper coordination of flexor and extensor muscle contractions, or more likely, from opposing inertial forces produced by the thigh segment that intensify at higher cadence levels [21,30]. The transition to hip power exertion at 2/8^th ^kp indicates a concurrence in both force and crank directions, and is most likely due to an increase in muscle forces resulting from a decrease in ergometer cadence [35]. The significant increase observed in HPO is consistent with Ericson's findings involving able-bodied subjects [23,33]. The higher cadences observed at 0/8^th ^and 1/8^th ^kp, may have produced thigh inertial forces that were larger in magnitude and opposite in direction to the force produced by the gluteus muscles. As a result, the hip joint absorbed power. At 2/8^th ^kp, the decrease in ergometer cadence resulted in a lower thigh inertial force. While the inertial force still opposed the direction of muscle force generation, the magnitude of the gluteus muscle force was sufficiently larger, promoting power exertion. The transition from power absorption to exertion at the hip with decreased cadence supports previous findings that suggest lower ergometer cadences to be favourable to higher muscle forces [35]. From a mechanical perspective, increased ergometer resistance incurred an increasingly larger moment about the hip. To compensate for this increase, the gluteal muscles increased force production, subsequently increasing HPO. Ergometer cadence and moment arm (pedal to hip) did not change significantly, therefore their influence on HPO is negligible. Since the hip was the only joint to exhibit an increase in power output in this exercise, the gluteal muscles may be the primary source of both power and continual force generation during FES-cycling in SCI subjects [21].

### Knee fatigue index

The index of fatigue developed in this study assesses a subject's capacity to pedal as described by their KPO. The knee was selected for the definition of this index due to its close proximity to the quadriceps and hamstring muscle groups. As demonstrated by the knee's regression equation, different combinations of stimulation and cadence may be examined to investigate riding conditions that may augment riding time and increase power output. The fatigue index value at 0/8^th ^kp, measured 90 seconds into active, unassisted pedaling was 1.6. This indicates initial power capacity at the knee to be 1.6 times greater than fatigue. The fatigue index decreased to 1.1 at 2/8^th ^kp, representing approximately a 30% decrease in the knee's power capacity within a 4 minute time span. These findings suggest that the present cycling protocol is not sufficient for a rider to gain the benefits of FES and thus raises speculation as to whether or not progressive resistance cycling is an appropriate protocol for SCI subjects. The index values derived from these five subjects, should be expanded and generalized to a larger population. If similar results emerge in future studies, this index may be useful within a clinical or experimental setting.

Mixing the results of both complete and incomplete SCI subjects was not considered a significant influence on the final outcome of this study. One of the incomplete SCI subjects produced results comparable to, and in some cases, better than complete SCI subjects. From this observation, it was determined that degree and frequency of ergometer use was a greater influence on the study results than extent of injury. Furthermore, using a mixed subject group of complete and incomplete SCI subjects is a truer representation of the FES-LCE user population. Variation existed within the subject group due to differences in age, time since injury (1.5 to 21 years), frequency and extent of cycling experience, as well as a likely difference in proportion of fast- to slow-twitch muscle fibers present in the stimulated muscle groups associated with variable atrophy. Previously, female SCI subjects have scored significantly higher on an endurance index than their male counterparts whereas no gender differences were noted between able-bodied control subjects in that particular study [31]. Other studies have suggested that the properties of muscle fiber in females may differ from males [36]. Future studies may investigate how the presently developed indices of fatigue may differ with gender. Additionally, alterations in stimulation timing and intensity could produce significantly different muscle synergies during FES cycling. Resulting joint power outputs and the subsequent development of a fatigue index would ultimately be affected. Therefore, the results of this study are valid for the presently employed stimulation protocol only. Additional research would be required to verify whether the present fatigue index is valid under different stimulation parameters.

## Conclusion

This study describes the influence of resistance on joint power output during FES cycle ergometry and the development of a fatigue index. While the objective of FES cycling ergometry is to help SCI subjects gain improved circulation, cardiovascular fitness and healthier muscle and bone, overcoming muscle fatigue is still an obstacle. Previous studies have found muscle force generation to be considerably weaker in SCI subjects than in able-bodied controls. Additionally, a decline in force production occurred earlier in SCI individuals [13,14]. The rapid increase in stimulation intensity and subsequent decrease in cadence and power output observed in this study suggest that the one-size-fits-all stimulation protocol may be one root cause of premature fatigue. Likewise, the use of a progressive resistance cycling therapy further contributes to fatigue onset. An index of fatigue was successfully developed, comparing knee power capacity during cycling with a predetermined value of fatigue. However, further studies should be conducted to determine if the power output predictors used here are valid for other stimulation protocols and subject groups.

## Competing interests

None of the authors have competing interest or financial relationship which may affect the results of this study.

## Authors' contributions

SAH carried out the ergometer testing, collected force and kinematic data, performed the statistical analysis and drafted the manuscript. PDF participated in the design and coordination of the study, data analysis, and drafting the manuscript. DJA helped in drafting the manuscript.
